# A rotation-translation invariant molecular descriptor of partial charges and its use in ligand-based virtual screening

**DOI:** 10.1186/1758-2946-6-23

**Published:** 2014-05-10

**Authors:** Francois Berenger, Arnout Voet, Xiao Yin Lee, Kam YJ Zhang

**Affiliations:** 1 Zhang Initiative Research Unit, Institute Laboratories, RIKEN, 2-1 Hirosawa, Wako, Saitama 351-0198, Japan

**Keywords:** RTI molecular descriptor, Partial charges, Ligand-based virtual screening, Spatial auto-correlation, Cross-correlation, Linear binning, ACPC

## Abstract

**Background:**

Measures of similarity for chemical molecules have been developed since the dawn of chemoinformatics. Molecular similarity has been measured by a variety of methods including molecular descriptor based similarity, common molecular fragments, graph matching and 3D methods such as shape matching. Similarity measures are widespread in practice and have proven to be useful in drug discovery. Because of our interest in electrostatics and high throughput ligand-based virtual screening, we sought to exploit the information contained in atomic coordinates and partial charges of a molecule.

**Results:**

A new molecular descriptor based on partial charges is proposed. It uses the autocorrelation function and linear binning to encode all atoms of a molecule into two rotation-translation invariant vectors. Combined with a scoring function, the descriptor allows to rank-order a database of compounds versus a query molecule. The proposed implementation is called ACPC (AutoCorrelation of Partial Charges) and released in open source. Extensive retrospective ligand-based virtual screening experiments were performed and other methods were compared with in order to validate the method and associated protocol.

**Conclusions:**

While it is a simple method, it performed remarkably well in experiments. At an average speed of 1649 molecules per second, it reached an average median area under the curve of 0.81 on 40 different targets; hence validating the proposed protocol and implementation.

## Background

Molecular similarity is a widely studied topic in chemoinformatics [1-3] and medicinal chemistry [4]. Molecular similarity is used in ligand-based virtual screening [5-8], SAR by catalog and to predict side effects. When hits are obtained in a drug discovery project, it is interesting to test similar derivatives in the hope that some will be more potent. Various molecular similarity measures have been developed [9]. Those include measures based on molecular descriptors [10], measures based on molecular fragments (such as MACCS) and measures based on graph matching (such as maximum common substructure searches [11-13]). For an extensive reference on molecular descriptors, cf. [10]. The DRAGON software [14] can compute thousands of such descriptors.

Electrostatics are one of the main driving forces of molecular recognition, along with steric complementarity, hydrogen bonding and hydrophobic interactions [15]. It is well known that there is complementarity in shape and electrostatics between a ligand molecule and its receptor protein. Molecules sharing similar electrostatics and shape are expected to bind to the same receptor. This principle has been used in ligand-based virtual screening to look for small molecules similar to known inhibitors and natural substrates [16-19]. The electrostatic potential of a molecule originates from the atomic partial charges. While the electrostatic reaches into the long range, the partial charges also contribute to the molecular recognition in the short range where they are the driving forces of polar interactions (hydrogen bonding and salt bridges) determining the specificity of recognition as well as the coordination of bridging waters between the receptor and ligand. Due to our recent study on the electrostatic similarity of small molecules with binding proteins [20], we were interested in the challenge of looking for active compounds while only exploiting the information contained in atomic coordinates and partial charges of a molecule.

The autocorrelation function has long been used in chemoinformatics, by several researchers and in various ways [21-27]. In 1980, Gilles Moreau and Pierre Broto proposed to use the autocorrelation function on the molecular graph to encode any property associated with atoms [21]. They subsequently used their autocorrelation descriptor in SAR studies [28,29]. The atom-type autocorrelation (ATAC) descriptors sum property values of atoms with given types [10]. Atom pairs [22] and Chemically Advanced Template Search (CATS) [30] are examples of such descriptors. The CATS2D descriptor [30], later extended to 3D [26], computes the topological cross-correlation of generalized atom types (hydrogen bond donor/acceptor, positively/negatively charged, lipophilic). The autocorrelation of partial charges of a 3D molecule encoded into histograms was studied [25]. Histograms of the autocorrelation of molecular surface properties were also used in partial least squares analysis to generate ligand-based 3D-QSARs [27], in PCA analysis [23], in Kohonen maps and in training neural networks for the prediction of biological activity [23,24].

A new molecular descriptor based on partial charges is proposed. It uses the autocorrelation function to encode all atoms of a molecule into two rotation-translation invariant vectors. The method is named ACPC (AutoCorrelation of Partial Charges). Compared to previous methods, neither using histograms nor explicitly computing the electrostatic potential field, but splitting the autocorrelation values based on their sign before applying linear binning [31] with a thin discretization step are essential traits of ACPC. Combined with a scoring function, it can rank-order a database of compounds versus a query molecule. The descriptor is solely based on the 3D distribution of partial charges, uses a single discretization parameter and provides good performance and speed. It was tested in a retrospective ligand-based virtual screening setting. At an average speed of 1649 molecules per second, it reached an average median Area Under the ROC^a^ Curve (AUC) of 0.81 on 40 different targets, outperforming several commonly used methods and making it a useful addition to the arsenal of ligand based virtual screening tools.

## Method

**Figure 1:**
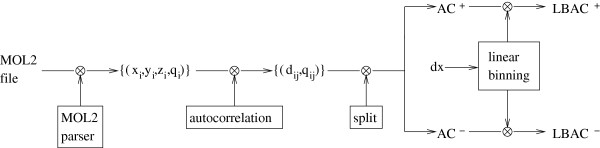
**Overview of theACPCmethod.** It encodes a 3D molecule into a rotation-translation invariant molecular descriptor based on partial charges and inter-atomic distances. The descriptor consists of two vectors separating the positive from the negative autocorrelation values. These vectors are discretized by linear-binning with discretization step *dx* in order to obtain the final pair of vectors (*LBAC*^+^ and *LBAC*^-^).

The point charge model of electrostatics is considered. In this model, each atom is a point in 3D space with an associated partial charge. The ACPC method first computes the autocorrelation of partial charges of all atoms in a molecule. Then, it separates the positive from negative values before applying linear binning. To score molecules, the sum of cross-correlations at lag zero is computed for corresponding vectors of the two molecules under consideration. A mathematical description follows and a graphical overview can be seen in Figure 1.

### Algorithm

Let *m* be a molecule with *A* atoms.

Let *i* = (*x*_*i*_,*y*_*i*_,*z*_*i*_,*q*_*i*_) be the atom at index i in *m* (1 < = *i* < = *A*), with 3D coordinates (*x*_*i*_,*y*_*i*_,*z*_*i*_) and partial charge *q*_*i*_.

Let *d*_*ij*_ be the Euclidean distance between atoms *i* and *j* of *m*.

Let *k* be the lag (an inter-atomic distance in fact).

Let $\delta_{kd_{\text{ij}}}$ be the Kronecker delta equal to one if *k* = *d*_*ij*_, zero otherwise.

Therefore, the autocorrelation of molecule *m* can be written as

$$\text{AC}(m,k)=\overset{A}{\underset{i=1}{\sum }}\overset{A}{\underset{j=1}{\sum }}q_{i}q_{j}\delta_{kd_{\text{ij}}}$$

Since in practice values of the autocorrelation vector for *k* = 0 are ignored and each atom pair is considered only once (*i*,*j* = *j*,*i*), the following formula is used:

$$\text{ACPC}(m,k)=\overset{A-1}{\underset{i=1}{\sum }}\overset{A}{\underset{j=i+1}{\sum }}q_{i}q_{j}\delta_{kd_{\text{ij}}}$$

Since the autocorrelation values can be positive or negative, they are split based on their sign before discretization, in order to avoid cancellation of values. This gives the two vectors *AC*^+^ and *AC*^-^.

$$\left({\text{AC}}^{+}(m,k),{\text{AC}}^{-}(m,k))=\text{sign\_split}(\text{ACPC}(m,k)\right)$$

such that *AC*^+^(*m*,*k*) is the subset of *ACPC*(*m*,*k*) where *q*_*i*_ ∗ *q*_*j*_ > = 0 and *AC*^-^(*m*,*k*) is the subset where *q*_*i*_ ∗ *q*_*j*_ < 0.

$$\text{ACPC}(m,k)={\text{AC}}^{+}(m,k)\cup {\text{AC}}^{-}(m,k)$$

To convert these two sets into vectors, linear binning [31] is used. Linear binning consists in selecting a discretization step (*dx*) for an axis, then linearly interpolating each raw data point’s contribution to the two neighboring points on the discretized axis. In our case, a raw data point is a pair of (*d*_*ij*_,*q*_*ij*_). Assuming that *d*_*ij*_ is between *x* and *z* (with *z* = *x* + *dx*) on the discretized axis, *q*_*ij*_ is separated into a contribution at *x* (called *c*_*x*_) and a contribution at *z* (called *c*_*z*_) by applying the formulae

$$c_{x}=q_{\text{ij}}*\frac{z-d_{\text{ij}}}{\text{dx}}\;\text{and}\;c_{z}=q_{\text{ij}}*\frac{d_{\text{ij}}-x}{\text{dx}}.$$

The result of linear binning is a vector containing the sum of all these contributions. Therefore, by applying linear binning, two rotation-translation invariant vectors encoding a molecule are obtained:

$$\begin{array}{l} \text{LBA}C^{+}(m,k,\text{dx})=\text{linbin}(AC^{+}(m,k),\text{dx})\\ \text{LBA}C^{-}(m,k,\text{dx})=\text{linbin}(AC^{-}(m,k),\text{dx}) \end{array}$$

By default, to score the similarity between two linearly-binned sign-split autocorrelation vectors, cross correlation at lag zero is used. Molecules being encoded in a rotation-translation invariant way, there is no need to scan for the lag which would yield the maximum cross-correlation. This maximum would be at lag zero for similar molecules. Since the goal is to assess the similarity of molecules, scanning the lag would be inappropriate.

Let *n* be another molecule.

Let *l*1 = *length*(*LBAC*^+^(*m*,*k*,*dx*)) and *l*2 = *length* (*LBAC*^+^(*n*,*k*,*dx*)) in *p* = *min*(*l*1,*l*2) and let *l*3 = *length*(*LBAC*^-^(*m*,*k*,*dx*)) and *l*4 = *length*(*LBAC*^-^(*n*,*k*,*dx*)) in *q* = *min*(*l*3,*l*4).

The cross-correlation at lag zero of molecule *m* with molecule *n* is $\text{CC}(m,n,\text{dx})=\sum_{k=0}^{p}\text{LBA}C^{+}(m,k,\text{dx})\text{LBA}C^{+}(n,k,\text{dx})+\sum_{k=0}^{q}\text{LBA}C^{-}(m,k,\text{dx})\text{LBA}C^{-}(n,k,\text{dx})$.

**Figure 2:**
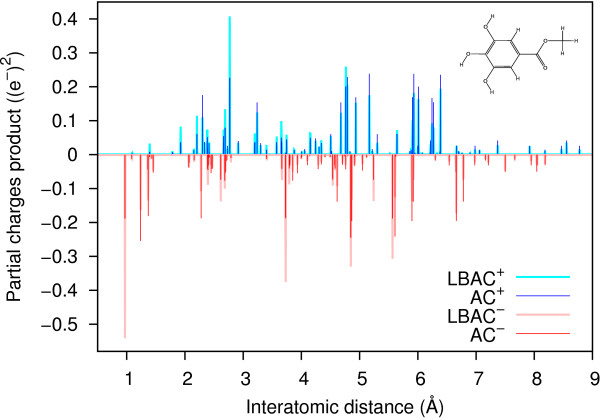
**Autocorrelogram.** In blue and red impulses: positive and negative values of the autocorrelation (AC^±^) of partial charges for the first ligand of the comt target. The ligand is depicted in 2D at the top right. The linearly binned versions (LBAC^±^) of the positive and negative parts of the autocorrelogram are the cyan and pink lines.

The spectrum obtained for one molecule, before and after linear binning can be seen in Figure 2. Differences in peak heights between *AC*^±^ and *LBAC*^±^ are due to linear binning. It is noteworthy that such spectra are not completely uninterpretable. Each Kronecker delta could be textually annotated by the names (from the MOL2 file for example) of the two atoms that give birth to it. Of course, such annotations would be crowded near *y* = 0 but readable for peaks which are standard deviations away from the mean.

### Software features

ACPC allows rank-ordering of a compound database against a query molecule. By default, ACPC filters out multiple conformers of the same molecule^b^ by keeping only the best scoring one. Molecular descriptors are computed and scored on the fly. Molecule names, scores and ranks are written to disk. A special program (acpc_big) is also provided to score large databases. In contrary to the default program, acpc_big executes in constant memory space but does not rank nor filter out multiple conformers. For tests on datasets with known active compounds (whose molecule names must be prefixed with the word “active”), the AUC is computed by the CROC package [32]. Several scoring functions are available (CC, Tanimoto, Tversky_ref_, Tversky_DB_). The following remaining features are optional. The computation can be parallelized on multicore computers using the Parmap library [33] for multiple queries on the same database. The filtering of multiple conformers can be turned off. The ROC and AUC calculations can be turned off. The ROC curve can be displayed on screen with Gnuplot [34]. The discretization step can be changed.

## Results and discussion

### Datasets

To evaluate ACPC, two different datasets were prepared. All ligands and decoys come from release two of the Directory of Useful Decoys (DUDr2) [35]. The DUD contains 2950 ligands for 40 different targets. Every ligand has 36 decoy molecules that are physically similar but topologically distinct. OMEGA v2.4.6 [36] was used to generate conformers. The first dataset (named “1conf”) contains a single, lowest energy conformer per molecule. It is further filtered to have unique SMILES. If several molecules have the same SMILES string as assigned by Open Babel [37], only the first encountered molecule was kept. This allows to easily detect and filter out multiple conformers of the same molecule later on, when one wants to keep only the highest scoring conformer. Finally, MOE [38] in combination with its default force-field (MMFF94x) was used to assign partial charges to molecules. As “1conf” is of reasonable size for each target, it is affordable to test each active of a given target in turn as a query, especially for the four software that run well in a cluster environment (ACPC, Pharao, Open Babel and Shape-it). The second dataset (named “25conf”) is the expansion of “1conf” by generating a maximum of 25 low energy conformers per molecule. As “25conf” is a much bigger dataset, each software is run only once with a single query per target. On “25conf”, it is feasible to test all the MOE fingerprints since a single query per target is performed. Cf. section “Availability and requirements” to download these datasets.

### Parametrization

The discretization parameter (*dx*) was setup using 20 randomly selected targets (from a total of 40). Then, for each selected target, only the first ligand in its ligands list was used to measure the AUC reached by ACPC. The *dx* parameter is introduced because of linear binning [31]. *dx* allows to balance the trade-off between the speed of the algorithm and the approximation error introduced by binning [31]. Small values of *dx* are important in our method since they counterbalance the information loss incurred by dimensionality reduction.

The default value proposed (*dx* = 0.005Å) gives a good compromise between speed and average AUC reached. *dx* values in the range 0.001 < = *dx* < = 0.009 are acceptable, but 0.001 consumes too much memory and values over 0.009 don’t perform as well in terms of AUC (Table 1).

**Table 1:** **Effect of the *dx * parameter**

***dx* (Å)**	**Average (AUCs)**	**Median (AUCs)**
0.005	0.73	0.75
0.01	0.69	0.71
0.05	0.68	0.70
0.1	0.67	0.70
0.5	0.66	0.66

Test protocol: 20 targets and five queries per target were randomly chosen on “1conf”.

Concerning the charge model used to assign partial charges; the default charge model from MOE (MMFF94x) was used in all experiments. For users with no access to MOE, the Gasteiger charge model [39] from Open Babel performs nearly as well. Other charge models available in Open Babel (QTPIE [40], QEq [41], MMFF94 [42,43]) perform worse and hence are not recommended for use with ACPC (cf. Table 2).

**Table 2:** Effect of the force field used to assign partial charges (OB stands for Open Babel)

**Force field**	**Average (AUCs)**	**Median (AUCs)**
MOE’s MMFF94x	0.77	0.84
OB’s Gasteiger	0.75	0.77
OB’s MMFF94	0.72	0.75
OB’s QEQ	0.70	0.72
OB’s QTPIE	0.69	0.74

Test protocol: 20 targets and five queries per target were randomly chosen on “1conf”.

Additional experiments were carried out retrospectively to confirm the choices of parameters. The test protocol was as follows: 20 targets and five queries per target were randomly selected on “1conf”. Then, the effect of the sign_split function, the *dx* parameter and the force field used to assign partial charges were measured in three distinct experiments. Those results are shown in Tables 1, 2 and 3. As can be seen from Table 3, the use of the sign_split function gave better AUCs. The finer discretization parameter gave the best AUCs compared to coarser ones (Table 1). Using the MMFF94x force field to assign partial charges gave the best AUCs compared to other force fields (Table 2).

**Table 3:** Effect of the sign_split function

	**With**	**Without**
Average (AUCs)	0.77	0.76
Median (AUCs)	0.83	0.75

Test protocol: 20 targets and five queries per target were randomly chosen on “1conf”.

### Validation protocol

ACPC was compared against a diverse set of freely available methods and to the molecular fingerprints available in MOE.

The open source software compared against are: 1) the Pharmacophore alignment and optimization tool Pharao [44], from Silicos-it [45] 2) the purely shape-based tool Shape-it, also from Silicos-it [46] and 3) the MACCS fingerprint as implemented in Open Babel [37].

Pharao is an open source software to align and score small molecules using pharmacophores. Pharao uses 3D Gaussians to represent pharmacophore features instead of the more common points and spheres model. Pharao’s performance has been demonstrated in virtual screening experiments and unsupervised clustering of small molecules [44]. Tversky_ref is recommended with Pharao to score compounds in virtual screening experiments [44].

Shape-it is a tool to align a reference molecule against a database of molecules. Shape-it uses 3D Gaussians to describe the molecular shape [47]. Shape-it can find the alignment of molecules which maximizes their volume overlap. Tversky_ref was used to score compounds with Shape-it, since it gave better results than Tanimoto or Tversky_db.

The MACCS fingerprint is a bit string registering the presence or absence of structural features (MACCS stands for Molecular ACCess System, originally developed by Molecular Design Limited, now Accelrys). In Open Babel, Tanimoto is used to score compounds with MACCS.

All ligand fingerprints available in MOE v2013.08 [38] have also been used. Some of the MOE fingerprints (TAD, TAT, TGT, TGD) can be traced back to the literature [48,49]. All MOE fingerprints use Tanimoto to score compounds, except ESshape3D and ESshape3d_HYD which use an inverse distance. If an abbreviated name is used in tables, it is given between parentheses below. 

• ESshape3D_HYD (ES3DH) is an eigenvalue spectrum shape fingerprint. It allows for comparison of 3D shapes made by hydrophobic heavy atoms of a molecule.

• ESshape3D (ES3D) is similar to ESshape3D_HYD but uses all heavy atoms instead of just hydrophobic ones.

• GpiDAPH3 (Gpi3) is a three points pharmacophore fingerprint calculated from the 2D molecular graph. Each atom is given one of eight atom types computed from three atomic properties (in pi system, is donor, is acceptor). Anions and cations are ignored.

• piDAPH3 (pi3) is similar to GpiDAPH3 but uses the molecule’s 3D conformation instead of the 2D molecular graph.

• piDAPH4 (pi4) is similar to piDAPH3 but considers quadruplets of pharmacophore features instead of triangles.

• TAD is a two points pharmacophore fingerprint calculated from the molecule’s 3D conformation. It considers pairs of pharmacophore features (donor, acceptor, polar, anion, cation, hydrophobic).

• TAT is similar to TAD but uses triangles instead of point pairs.

• TGD is similar to TAD but uses the 2D molecular graph instead of the 3D conformation.

• TGT is similar to TGD but uses triangles instead of point pairs.

PAR [50] was used to accelerate some experiments by parallelizing their execution on a multicore computer.

### Performance

As a reminder about reading AUC values from ROC curves: *AUC* = 0.5 is the performance of a random method. An AUC score of less than 0.5 means that a method perform worse than random. All rationally engineered methods are expected to perform significantly above 0.5 in terms of AUC.

**Figure 3:**
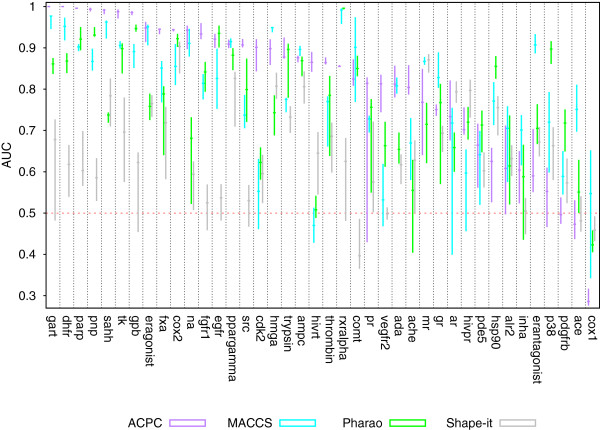
**1st, 2nd and 3rd quartile plots forACPC, MACCS, Pharao and Shape-it on all queries of the “1conf” dataset.** For clarity, targets are sorted on the x axis by decreasing median value obtained by ACPC. The red dotted horizontal line at AUC = 0.5 indicates random performance.

**Figure 4:**
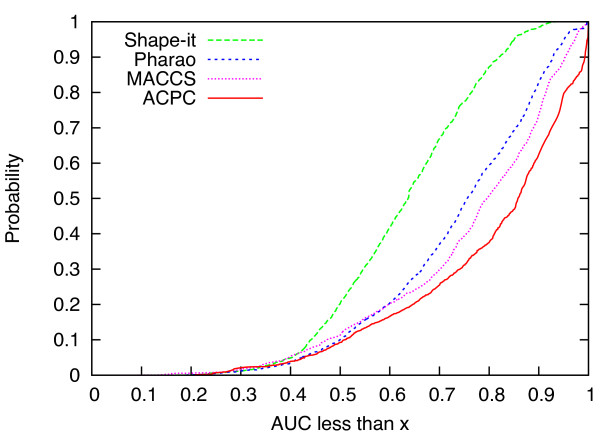
**Cumulative distribution functions for Shape-it, Pharao, MACCS and** **ACPC** **on all queries of the “1conf” dataset.**

Test results using all possible queries on the “1conf” dataset are shown in Figure 3 as a per target quartile plot and in Table 4 as median AUCs, while Figure 4 gives an aggregated overview.

**Table 4:** Median AUCs on the “1conf” dataset

**Target (L/D)**	**ACPC**	**Pharao**	**MACCS**	**Shape-it**
ace (49/1753)	0.47	0.55	***0.75***	0.48
ache (106/3711)	***0.80***	0.56	0.67	0.63
ada (37/844)	***0.81***	0.65	***0.81***	0.62
alr2 (23/939)	0.61	0.61	***0.70***	0.63
ampc (21/767)	0.88	0.87	***0.90***	0.81
ar (74/2709)	0.73	0.66	0.72	***0.79***
cdk2 (58/1866)	***0.90***	0.62	0.55	0.60
comt (10/425)	0.82	0.85	***0.90***	0.40
cox1 (25/885)	0.29	0.42	***0.55***	0.46
cox2 (411/12281)	***0.94***	0.92	0.85	0.90
dhfr (405/7418)	***1.00***	0.87	0.95	0.62
egfr (458/14449)	0.92	***0.94***	0.83	0.54
er+ (67/2387)	***0.95***	0.76	***0.95***	0.77
er- (39/1330)	0.59	0.70	***0.91***	0.67
fgfr1 (120/4305)	***0.93***	0.84	0.81	0.53
fxa (146/4969)	***0.94***	0.79	0.85	0.72
gart (31/845)	***1.00***	0.86	0.98	0.68
gpb (50/2072)	***0.99***	0.95	0.89	0.62
gr (78/2803)	0.75	0.77	***0.83***	0.69
hivpr (57/1797)	0.70	0.72	0.60	***0.80***
hivrt (41/1433)	***0.86***	0.51	0.47	0.65
hmga (35/1362)	0.90	0.74	***0.95***	0.81
hsp90 (25/918)	0.62	***0.85***	0.77	0.76
inha (79/3131)	0.60	0.59	***0.70***	0.51
mr (14/561)	0.77	0.71	***0.87***	***0.87***
na (49/1826)	***0.94***	0.68	0.91	0.59
p38 (366/8722)	0.55	***0.90***	0.72	0.66
parp (35/1296)	***1.00***	0.92	0.90	0.60
pde5 (76/1955)	0.66	***0.71***	0.64	0.60
pdgfrb (169/5560)	0.50	***0.72***	0.59	0.57
pnp (30/962)	***0.99***	0.93	0.87	0.59
ppar *γ* (82/2635)	0.91	0.88	***0.92***	0.83
pr (27/989)	***0.81***	0.76	0.73	0.57
rxr *α* (20/724)	0.85	***1.00***	0.99	0.62
sahh (32/1250)	***0.99***	0.74	0.96	0.78
src (159/5904)	***0.91***	0.80	0.74	0.53
thrombin (67/2308)	***0.86***	0.79	0.77	0.69
tk (22/860)	***0.99***	0.90	0.91	0.70
trypsin (46/1565)	0.88	***0.90***	0.78	0.73
vegfr2 (77/2701)	***0.81***	0.66	0.53	0.50
Average	0.81	0.77	0.79	0.65
Median	0.86	0.77	0.82	0.63
|Best method|	20	7	13	3

For each of the 40 targets, each ligand in the ligands list was used in turn as the query. On each line, the maximum value is underlined and in bold font. L = number of ligands; D = number of decoys.

In Figure 3, three classes are distinguishable. Class 1: ACPC is far in front. Class 2: ACPC’s performance ties or significantly overlaps with at least one other method. Class 3: ACPC is outperformed by at least one other method. Class 1 targets (17 cases): gart, dhfr, parp, pnp, sahn, tk, gbp, fxa, cox2, fgfr1, src, cdk2, hivrt, thrombin, pr, vegfr2 and ache. Class 2 targets (9 cases): tie in eragonist, na, egfr, ppargamma, trypsin, ampc, ada, ar and pde5. Class 3 targets (13 cases): hmga, rxralpha, comt, mr, gr, hivpr, hsp90, alr2, inha, erantagonist, p38, pdgfrb, ace. Cox1 is classified as an exception as no method perform well on this target: ACPC, Pharao and Shape-it all have median AUCs below 0.5 and MACCS has a huge standard deviation with a mean not far from 0.5. By looking at the cumulative distribution function (CDF) from experiments on “1conf” in Figure 4, such statements can directly be read: the probability for ACPC to have an AUC less than 0.5 is about 10%. All other software have a higher probability to have an AUC less than 0.5. By looking at the CDF, the previous statement is seen to hold for ACPC for any *AUC* > = 0.41. The best performance is reached 20 times by ACPC, 13 times by MACCS, seven times by Pharao and three times by Shape-it.

Test results on the “25conf” dataset are shown in Table 5. In terms of average AUC reached, the best method is ACPC (0.78) followed by GpiDAPH3 (0.72) then MACCS (0.7). The best AUC is reached 17 times by ACPC, five times by Pharao and four times by the MOE fingerprints TAD and TAT. On average, the GpiDAPH3 fingerprint from MOE is the second best method on this dataset. In order to confirm the stability of the test results on the “25conf” dataset using a single query, ACPC was run with all the active ligands as queries. The resulting average AUCs are shown in column ACPC_25*c*_ in Table 5. For comparison, the average AUCs from the “1conf” dataset are also calculated and shown in columns ACPC_1*c*_ in Table 5. The performance of ACPC on “25conf” using a single query is stable, since the average AUCs are comparable to that using all the actives as queries. Moreover, the performance of ACPC using either a single query or all actives as queries is similar to that from the “1conf” dataset.

**Table 5:** Average AUCs on the “25conf” dataset

**target (L/D)**	**ACPC1**_***c***_	**ACPC25**_***c***_	**ACPC**	**Pharao**	**MACCS**	**Shape-it**	**ES3DH**	**ES3D**	**Gpi3**	**pi3**	**pi4**	**TAD**	**TAT**	**TGD**	**TGT**			
ace (1140/43653)	0.49	0.48	0.45	0.58	0.68	0.57	0.34	0.44	***0.72***	0.60	0.62	0.61	0.57	0.49	0.60			
ache (2450/89138)	0.80	0.80	0.80	0.69	0.72	0.69	0.48	0.28	0.81	***0.85***	***0.85***	0.66	0.75	0.60	0.74			
ada (876/18697)	0.82	0.81	0.78	0.66	0.80	0.64	0.71	0.42	0.81	0.75	0.74	0.84	0.82	0.85	***0.87***			
alr2 (348/16063)	0.60	0.59	0.47	***0.74***	0.42	0.52	0.35	0.33	0.54	0.66	0.64	0.32	0.24	0.29	0.44			
ampc (507/15069)	0.84	0.83	0.89	0.86	0.88	0.71	0.82	0.59	0.87	0.87	***0.90***	0.75	0.83	0.84	0.75			
ar (363/42557)	0.75	0.73	0.72	0.67	0.80	0.75	0.68	0.44	0.71	0.76	0.71	***0.81***	0.66	0.80	0.67			
cdk2 (1138/43682)	0.88	0.88	***0.93***	0.62	0.49	0.54	0.42	0.50	0.66	0.68	0.67	0.34	0.41	0.34	0.46			
comt (124/7148)	0.83	0.81	0.80	***0.81***	0.77	0.65	0.38	0.34	0.76	0.62	0.75	0.43	0.46	0.46	0.53			
cox1 (422/15672)	0.30	0.29	0.27	0.55	0.51	0.46	0.47	0.38	0.50	0.40	0.48	0.49	0.61	0.50	***0.64***			
cox2 (6483/270311)	0.94	0.94	***0.87***	0.46	0.15	0.16	0.62	0.52	0.22	0.26	0.33	0.49	0.50	0.65	0.74			
dhfr (9550/166944)	1.00	1.00	***1.00***	0.94	0.96	0.56	0.63	0.46	0.98	0.93	0.98	0.99	***1.00***	0.98	0.91			
egfr (9964/337283)	0.91	0.89	0.90	***0.96***	0.78	0.57	0.49	0.25	0.89	0.69	0.89	0.50	0.66	0.46	0.53			
er+ (289/39507)	0.92	0.91	0.94	0.81	0.95	0.63	0.26	0.38	0.76	0.89	0.68	***0.96***	***0.96***	***0.96***	***0.96***			
er- (975/32961)	0.62	0.60	0.71	0.75	0.89	0.66	0.29	0.40	0.83	0.84	0.69	***0.95***	0.92	***0.95***	0.92			
fgfr1 (2360/106612)	0.93	0.94	***0.87***	0.54	0.55	0.63	0.61	0.41	0.79	0.59	0.49	0.37	0.49	0.36	0.50			
fxa (3647/123379)	0.89	0.89	***0.64***	0.61	0.48	0.40	0.39	0.42	0.42	0.40	0.44	0.24	0.42	0.29	0.30			
gart (775/20938)	1.00	1.00	***1.00***	0.94	0.98	0.69	0.89	0.89	0.97	0.97	0.98	0.98	0.98	0.96	0.98			
gpb (845/44604)	0.97	0.96	***0.87***	0.23	0.68	0.42	0.24	0.29	0.34	0.16	0.32	0.18	0.20	0.17	0.16			
gr (553/56086)	0.73	0.72	0.75	0.75	***0.86***	0.55	0.50	0.48	0.79	0.82	0.85	0.64	0.67	0.62	0.60			
hivpr (1404/44909)	0.72	0.72	0.79	0.73	0.47	0.74	0.63	0.56	0.44	0.42	0.53	0.92	***0.93***	0.90	0.92			
hivrt (822/32688)	0.86	0.86	***0.82***	0.47	0.43	0.28	0.55	0.47	0.47	0.57	0.51	0.31	0.39	0.39	0.34			
hmga (814/33684)	0.89	0.89	0.87	0.83	0.94	0.85	0.57	0.53	0.94	0.93	0.94	***0.99***	0.98	0.97	0.98			
hsp90 (572/20983)	0.58	0.56	0.67	0.81	0.70	0.58	0.84	0.59	***0.93***	0.89	0.79	0.52	0.66	0.58	0.61			
inha (1782/71064)	0.58	0.58	0.59	0.48	***0.78***	0.48	0.24	0.30	0.33	0.35	0.36	0.41	0.66	0.49	0.71			
mr (79/10177)	0.73	0.70	0.54	0.18	0.69	0.72	***0.82***	0.60	0.75	0.65	0.48	0.49	0.43	0.46	0.58			
na (987/44278)	0.92	0.92	***0.93***	0.79	0.91	0.39	0.37	0.33	0.87	0.79	0.84	0.72	0.88	0.74	0.72			
p38 (7014/186992)	0.54	0.51	0.57	***0.89***	0.76	0.69	0.62	0.38	0.87	0.82	0.88	0.48	0.60	0.38	0.59			
parp (245/12871)	0.97	0.97	***0.99***	0.93	0.93	0.73	0.55	0.53	0.97	0.93	0.96	0.83	0.89	0.74	0.89			
pde5 (1751/48431)	0.66	0.65	***0.75***	0.67	0.68	0.69	0.39	0.44	0.67	0.62	0.67	0.31	0.35	0.36	0.34			
pdgfrb (3206/134591)	0.51	0.52	0.49	0.46	0.49	0.61	0.57	0.49	***0.62***	0.44	0.42	0.34	0.46	0.36	0.50			
pnp (560/16694)	0.99	0.98	***0.99***	0.95	0.90	0.63	0.36	0.52	0.90	0.76	0.91	0.82	0.86	0.87	0.91			
ppar *γ* (2010/65783)	0.91	0.90	0.88	0.89	0.89	0.70	0.21	0.25	0.92	**0.94**	0.92	0.86	***0.94***	0.76	0.86			
pr (178/15966)	0.70	0.70	0.37	0.37	0.48	0.67	0.30	0.19	0.73	***0.76***	0.56	0.61	0.66	0.64	0.63			
rxr *α* (392/17243)	0.77	0.77	0.86	***1.00***	0.99	0.70	0.53	0.49	0.95	0.97	0.99	0.94	0.95	0.99	0.90			
sahh (586/25622)	0.98	0.98	0.96	0.57	***0.97***	0.71	0.78	0.65	0.96	0.93	0.95	0.89	0.95	0.90	0.96			
src (2945/145751)	0.90	0.90	***0.81***	0.51	0.47	0.65	0.69	0.53	0.60	0.43	0.36	0.30	0.41	0.26	0.40			
thrombin (1576/57564)	0.87	0.88	***0.95***	0.74	0.51	0.65	0.54	0.58	0.65	0.48	0.36	0.71	0.68	0.62	0.66			
tk (379/15017)	0.98	0.98	***0.98***	0.84	0.92	0.54	0.47	0.37	0.89	0.86	0.87	0.86	0.86	0.85	0.89			
trypsin (1128/39065)	0.90	0.90	***0.98***	0.64	0.29	0.45	0.46	0.28	0.30	0.29	0.41	0.72	0.80	0.69	0.81			
vegfr2 (1604/66098)	0.79	0.78	***0.67***	0.47	0.42	0.44	0.32	0.24	0.57	0.54	0.48	0.37	0.41	0.33	0.38			
Average	0.80	0.79	0.78	0.68	0.70	0.59	0.51	0.44	0.72	0.68	0.68	0.62	0.67	0.62	0.67			
Median	0.85	0.84	0.81	0.71	0.74	0.63	0.49	0.44	0.76	0.72	0.69	0.62	0.66	0.62	0.67			
|Best method|	N/A	N/A	17	5	3	0	1	0	3	3	2	4	4	2	3			

For each of the 40 targets, the query was the last ligand in the ligands list of each target. For each target, the maximum AUC reached is underlined and in bold font. L = number of ligands; D = number of decoys. The ACPC_1*c*_ and ACPC_25*c*_ columns were computed and added for comparison. ACPC_1*c*_ (resp. ACPC_25*c*_) shows the average AUC reached when using all active ligands as queries for ACPC on “1conf” (resp. “25conf”). Their |best method | cells were not filled in since they concern different experiments than other columns.

**Figure 5:**
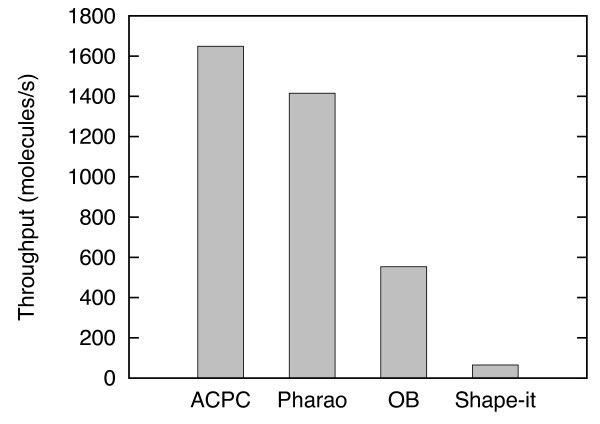
**Average processing speed of** **ACPC, Pharao, Open Babel (OB) and Shape-it on the target with the most ligands and decoys (egfr).**

Speeds of the software ran on “1conf” are shown in Figure 5. Speed tests were performed on one core of a 2.4 GHz Intel Xeon workstation with 12 GB of RAM running Ubuntu Linux 12.04. Speed measurements were done on the egfr target, since it has the most ligands and decoys. Reported numbers were averaged over three runs. Only scoring was performed by each method, no ranking or filtering of compounds was done. ACPC and Shape-it screen a database of compounds read from a MOL2 file. Open Babel reads compounds from a SMILES file. Pharao reads compounds from a.phar file (its own text format for pharmacophore features). ACPC processed 1649 molecules per second, Pharao 1416, Open Babel 553 and Shape-it 65.

The scaffold diversity among actives in the top 10 to 50 ranked molecules was also investigated for cox2 and egfr, the two targets with the most ligand clusters (44 and 40 respectively). Andrew Good’s clustering analysis of DUD ( http://dud.docking.org/clusters/) was used to assign each active to a cluster via reduced graphs [51]. Ten random queries were used on each target then cluster of actives in the top 10 to 50 molecules were analyzed for each method (Table 6). While the number of distinct clusters of actives found in the top 10 molecules is somewhat comparable for ACPC, Pharao, MACCS and Shape-it; the rate at which new clusters of actives are discovered by ACPC doesn’t increase as fast as other methods. But this is to be expected; pharmacophores (Pharao) are a powerful way of generalizing atom types while shape (Shape-it) is an even more permissive representation of molecules.

**Table 6:** Average number of distinct clusters found for active molecules among the top N ranked molecules for cox2 and egfr in “1conf” using 10 random queries

**Target**	**cox2**				**egfr**			
**N**	**ACPC**	**Pharao**	**MACCS**	**Shape-it**	**ACPC**	**Pharao**	**MACCS**	**Shapeit**
10	2.3	2.2	2.8	2.3	0.9	2.4	1.4	2.2
20	2.7	3.3	3.9	3.5	1.1	3.0	2.4	3.1
30	3.1	4.4	4.5	4.6	1.3	4.4	2.9	3.6
40	3.4	4.9	5.1	5.4	1.5	5.2	3.5	3.9
50	3.5	5.8	5.4	6.3	2.0	5.8	4.7	4.1

Early during the development of the method, scoring the similarity of descriptors with Pearson’s r and Spearman’s rank order correlation coefficient [52] was tried. Later on, distance metrics for continuous variables were tried (some equations can be found in [26,44]): the Tanimoto coefficient, Tversky_ref, Tversky_db and $\frac{1}{1+d}$ where *d* is the Manhattan distance or the Euclidean distance. None of these performed better than cross-correlation in tests on small random partitions of the DUD (20 queries chosen randomly across all ligands and targets).

ACPC’s good performance might be explained by the fact that all atoms of a molecule are considered at the same time and all intra molecular distances are handled in the same way. While atom centers partially encode the shape, partial charges encode some of the recognition features (eg. hydrogen bond acceptors and donors). ACPC measures the global similarity of two molecules in terms of intra molecular vectors and partial charges. The high number of molecules per second the method can process is a direct benefit from its simplicity and reliance on a strong mathematical property: being rotation-translation invariant. In ACPC, molecules do not need to be optimally superposed before scoring and the electrostatic potential field is not computed.

### Recommended usage

ACPC was designed to be rotation and translation invariant. However, ACPC is not invariant to the conformer of a molecule, neither to the charge model that was used to assign partial charges (Table 2). ACPC is also sensitive to the choice of the *dx* parameter (Table 1). Hence, the following protocol has been validated: the query molecule(s) *and* the database to screen must be prepared in the same way. The same software with same parameters must be used to assign partial charges and generate conformers for *all* molecules.

### Limitations

Our method, by construction, cannot distinguish a molecule *m* from its enantiomer. If *n* is a perfect mirror image of *m* and *m*^′^ is a copy of *m* with all partial charges reversed (sign flipped) and *n*^′^ is a copy of *n* with all partial charges reversed, then they cannot be distinguished.

$$\begin{array}{ll} \;\text{CC}(m,m,\text{dx}) & =\text{CC}(m,m^{\prime },\text{dx})=\text{CC}(m,n,\text{dx})\\ & =\text{CC}(m,n^{\prime },\text{dx}) \end{array}$$

Also, since the method is purely based on partial charges, it should perform poorly if the recognition of a binding site by a query ligand is not mostly driven by electrostatics. This should be the case for binding sites that are predominantly non-polar.

While release two of the DUD [35] was used to prepare datasets, it has known shortcomings. DUD was initially created as a benchmark set for molecular docking but, like some previous authors [19,53,54], we use it to test ligand-based virtual screening methods. If some ligands L1 and L2 of the same target are targeting different sub-pockets of the binding site, it is incorrect to use one of them (in a ligand-based approach) to find the other. Another problem previously noticed by other authors [55] is that DUD decoys are only supposed to be inactive. If tested experimentally, some decoys may be found to be active. In future studies, DUD Enhanced, a more recent version of DUD [56] which overcomes some of its previous drawbacks and includes more targets might be used.

Potential users should keep in mind that there is no silver bullet for *in-silico* drug discovery. ACPC is no exception. From previously shown results, MACCS or Pharao or some of the MOE fingerprints are seen to perform better than ACPC on several targets. Also, Pharao, MACCS and Shape-it seem to promote scaffold diversity of actives earlier in the ranked list of compounds (Table 6).

### Upcoming features

The following features are under consideration for future releases of ACPC. On the purely technical side, a GPU-based version of the tool is doable [57] to reach higher throughput. Combined with a metric distance, clustering compounds databases would then become computationally tractable. Other interesting topics would require more research and experiments, such as the automatic creation of a consensus query from a set of known actives, investigating other orthogonal feature spaces such as atomic radii, solvent accessible areas and per atom hydrophobic contribution, to increase the discriminative power of the method. The choice and parametrization of suitable kernel functions for use in Kernel Density Estimates (KDE) is also an interesting direction that could result in reduced sensitivity to the discretization parameter and probably better AUCs (at the cost of heavier computation). With KDE, the method could probably be extended to cluster binding sites based on the partial charges of their surface atoms.

## Conclusions

We revisited the family of rotation-translation invariant molecular descriptors and proposed a new, simple but powerful encoding of the autocorrelation of partial charges of a 3D molecule. Our implementation is fast and displays good performance in retrospective ligand-based virtual screening experiments. ACPC should be a useful tool for ligand-based virtual screening. ACPC is open source, freely available and automatically installable as an OPAM [58] package. Requests and contributions from users are welcome.

## Availability and requirements

**Project Name:**ACPC

**Project home page:**http://www.riken.jp/zhangiru/software.html

**Operating system:** Linux

**Programming language:** OCaml http://ocaml.org

**Other requirements:** the OCaml Package Manager (OPAM) http://opam.ocaml.org/

**Dataset (352 MB):**http://www.riken.jp/zhangiru/software/DUD_ACPC_1.0_validation.tar.xz The CROC Python package (optional) http://pypi.python.org/pypi/CROC/ Gnuplot [34] (optional) http://www.gnuplot.info/

**License:** BSD

**Any restrictions to use by non-academics:** None

## Endnotes

^a^Receiver Operating Characteristic.

^b^They should have the same molecule name.

## Competing interests

The authors declare that they have no competing interests.

## Authors’ contributions

FB designed the method, wrote the software, ran experiments and prepared figures and tables. XYL, FB and AV prepared the datasets. FB, AV, XYL and KYJZ analyzed the results, wrote, read and approved the final manuscript.
